# Capu and Spire Assemble a Cytoplasmic Actin Mesh that Maintains Microtubule Organization in the *Drosophila* Oocyte

**DOI:** 10.1016/j.devcel.2007.09.003

**Published:** 2007-10-09

**Authors:** Katja Dahlgaard, Alexandre A.S.F. Raposo, Teresa Niccoli, Daniel St Johnston

**Affiliations:** 1 The Wellcome Trust/Cancer Research UK Gurdon Institute and The Department of Genetics, University of Cambridge, Tennis Court Road, Cambridge CB2 1QN, UK

**Keywords:** CELLBIO, DEVBIO

## Abstract

Mutants in the actin nucleators Cappuccino and Spire disrupt the polarized microtubule network in the *Drosophila* oocyte that defines the anterior-posterior axis, suggesting that microtubule organization depends on actin. Here, we show that Cappuccino and Spire organize an isotropic mesh of actin filaments in the oocyte cytoplasm. *capu* and *spire* mutants lack this mesh, whereas overexpressed truncated Cappuccino stabilizes the mesh in the presence of Latrunculin A and partially rescues *spire* mutants. Spire overexpression cannot rescue *capu* mutants, but prevents actin mesh disassembly at stage 10B and blocks late cytoplasmic streaming. We also show that the actin mesh regulates microtubules indirectly, by inhibiting kinesin-dependent cytoplasmic flows. Thus, the Capu pathway controls alternative states of the oocyte cytoplasm: when active, it assembles an actin mesh that suppresses kinesin motility to maintain a polarized microtubule cytoskeleton. When inactive, unrestrained kinesin movement generates flows that wash microtubules to the cortex.

## Introduction

The main body axes of *Drosophila* are established during stages 7–9 of oogenesis when the oocyte microtubule (MT) cytoskeleton is reorganized to direct the asymmetric localization of *bicoid* (*bcd*), *oskar* (*osk*), and *gurken* mRNAs (Johnstone and Lasko, 2001). At stage 7 of oogenesis, an unknown signal from the posterior follicle cells induces the disassembly of a microtubule-organizing center at the posterior of the oocyte, while new MTs nucleate from the anterior-lateral cortex with their plus ends extending toward the posterior pole (Cha et al., 2001; Theurkauf et al., 1992). This results in the formation of an anterior-to-posterior gradient of MTs that directs the localization of *bcd* and *osk* mRNAs to the anterior and posterior poles of the oocyte, respectively, where they act to determine the anterior-posterior axis of the embryo (St Johnston, 2005). The polarized MT cytoskeleton is also required for the migration of the oocyte nucleus from the posterior of the oocyte to a point at the anterior margin, and this defines the dorsal-ventral axis by directing the localization of *gurken* mRNA to one side of the nucleus, where Gurken protein is secreted to induce dorsal follicle cell fates (Neuman-Silberberg and Schüpbach, 1993; van Eeden and St Johnston, 1999).

The organization of the MTs changes during stage 10B, and they form parallel arrays around the cortex of the oocyte that drive a fast unidirectional movement of the oocyte cytoplasm, called ooplasmic streaming (Theurkauf et al., 1992). Ooplasmic streaming requires the plus-end-directed MT motor, Kinesin, suggesting that the flows are generated by kinesin-dependent transport of organelles or vesicles (Palacios and St Johnston, 2002). The cytoplasm is also in motion in oocytes from stages 8−10A, but these movements are slower and uncoordinated and have been named ooplasmic seething.

The polarized organization of the MTs at mid-oogenesis requires the function of *par-1* and *capu* groups of genes. In mutants in the former group, which comprises *par-1*, *lkb-1*, and *14-3-3ɛ*, the MTs appear to be nucleated all around the oocyte cortex, with their plus ends in the center (Benton et al., 2002; Martin and St Johnston, 2003; Shulman et al., 2000). As a consequence, *osk* mRNA is mislocalized to a dot in the center of the oocyte, while *bcd* mRNA spreads from the anterior around most of the cortex. However, the localization of *gurken* mRNA is wild-type in these mutants. The polarity signal from the follicle cells induces the actin-dependent localization of PAR-1 to the posterior cortex of the oocyte, suggesting that asymmetric PAR-1 activity plays a key role in the polarization of the oocyte MT cytoskeleton (Doerflinger et al., 2006).

Mutants in *cappuccino* (*capu*), *chickadee* (*chic*), and *spire* produce a distinct phenotype, in which the MTs form prominent arrays around the oocyte cortex during stages 8–10 and MT plus-end markers no longer localize to the posterior pole (Clark et al., 1994; Emmons et al., 1995; Manseau et al., 1996; Theurkauf, 1994; Wellington et al., 1999). These mutants also cause premature streaming of the oocyte cytoplasm, which resembles the cytoplasmic streaming seen in wild-type oocytes after stage 10B (Manseau et al., 1996; Theurkauf, 1994). As a result, both *osk* and *gurken* mRNAs are mislocalized, leading to abdominal defects in the embryo and ventralized eggs, although the localization of *bcd* mRNA is unaffected (Manseau and Schupbach, 1989).

Actin-depolymerizing drugs produce identical MT and premature cytoplasmic streaming phenotypes to *capu*, *chic*, and *spire* mutants, indicating that actin is required for the correct organization of the MT cytoskeleton (Manseau et al., 1996). Consistent with this, all three genes encode regulators of the actin cytoskeleton. Chickadee is *Drosophila* Profilin, which binds free G-actin protein to regulate actin dynamics (Cooley et al., 1992; Yarmola and Bubb, 2006); Spire is the founding member of a new family of actin nucleation factors that nucleate filaments from their pointed ends (Quinlan et al., 2005; Wellington et al., 1999); Capu is a member of the Formin family of proteins, which also nucleate actin filaments, but in this case from their barbed ends (Emmons et al., 1995; Kovar and Pollard, 2004; Pruyne et al., 2002; Sagot et al., 2002).

Although effects of actin depolymerization strongly suggest that actin plays a key role in the organization of the oocyte MT cytoskeleton, it is not clear which population of F-actin in the oocyte is responsible for this effect, or how Capu, Spire, and Profilin participate in the interaction between actin and MTs. One possibility is that Capu, Profilin, and Spire regulate MTs by directing the posterior recruitment of PAR-1, since they have been proposed to play a role in the organization of cortical actin, which is required for PAR-1 localization (Doerflinger et al., 2006; Rosales-Nieves et al., 2006). This cannot account for all of the effects of the *capu* group mutants, however, since they produce a different phenotype from *par-1* mutants. An alternative possibility is suggested by experiments showing that formin-related proteins can control the positioning or stability of MT plus ends. Bni1p is required for spindle positioning during early metaphase in budding yeast, through the recruitment of the plus ends of astral MTs to the bud tip. Bni1p localizes to the emerging bud tip and nucleates unbranched actin filaments (Evangelista et al., 2002). The myosin, Myo2p, then transports the MT plus ends along these actin cables to the bud tip, through its linkage to the plus-end-binding protein, Kar9p (Hwang et al., 2003). In contrast, the mouse formin mDia1 acts independently of actin to stabilize MT plus ends at the leading edge of migrating NIH 3T3 cells, through a pathway that involves the inhibition of GSK3β and the plus-end-binding proteins, EB1 and APC (Eng et al., 2006; Palazzo et al., 2001). Thus, Capu may function in a similar way to either Bni1 or mDia to recruit or stabilize MT plus ends at the posterior of the oocyte.

Rosales-Nieves et al. (2006) have recently proposed a different model for the function of Capu and Spire, in which they act not as actin nucleators but as crosslinkers between MTs and cortical actin. The *spire* locus encodes multiple isoforms, including two short forms, Spire D and Spire C, that encompass the N-terminal and C-terminal halves of the longest isoform, respectively. Spire D contains the KIND domain and the 4 WH2 domains that have been shown to nucleate actin in vitro and when transiently expressed in mouse fibroblasts, whereas Spire C includes an mFYVE domain and a JNK-binding site (Kerkhoff et al., 2001; Otto et al., 2000; Quinlan et al., 2005; Wellington et al., 1999). In binding studies with tubulin and actin in vitro, both Capu and Spire C induced the bundling of actin with MTs (Rosales-Nieves et al., 2006). In contrast, Spire D nucleated F-actin in vitro but did not interact with MTs and inhibited the actin/MT crosslinking activity of Capu and Spire C. This has led to the proposal that Capu and Spire C repress the cortical bundling of MTs and premature cytoplasmic streaming by crosslinking the MTs to the cortical actin, whereas Spire D is a negative regulator of this process (Rosales-Nieves et al., 2006).

To distinguish between the different models for the function of Capu and Spire, we expressed various domains of each protein in wild-type and mutant egg chambers in order to analyze their subcellular localizations and their effects on actin, MTs, and cytoplasmic streaming in vivo. Our results indicate that neither the cortical localization nor the MT-binding activity of Capu and Spire is required for their function. Instead, we show that Capu and Spire act to assemble a dynamic actin mesh in the oocyte cytoplasm.

## Results

### The Cappuccino Pathway Is Not Required for Polarization of the Oocyte Cortex

**Figure 1:**
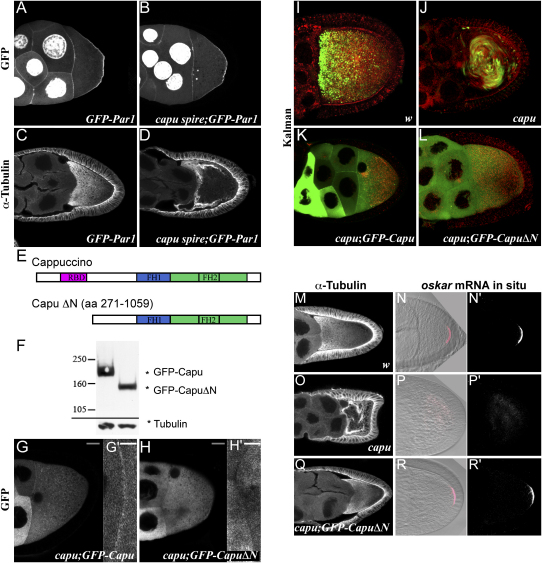
The Cappuccino Pathway Is Not Required for Polarization of the Oocyte Cortex (A and B) GFP-PAR1 linker domain localization in a wild-type stage 9 oocyte (A) and in a *capu^RK^ spire^RP^* oocyte (B). (C) α-Tubulin staining of a wild-type stage 9 egg chamber. (D) α-Tubulin staining of a *capu^RK^ spire^RP^* egg chamber. (E) Domain structure of full-length Cappuccino (PA) and the CapuΔN construct showing the RhoA-binding domain (RBD, amino acids 125–250); the formin homology domain 1 (FH1, amino acids 485–646); and the formin homology domain 2 (FH2, amino acids 796–867). (F) Western blot showing the expression levels of GFP-Capu and GFP-CapuΔN detected with an α-GFP antibody. α-Tubulin was used as loading control. (G and H) Confocal images of GFP-Capu (G) or GFP-CapuΔN (H) in live stage 9 *capu^G7^/Df(2L)ed^SZ1^* egg chambers. The gray scale bars indicate 20 μm. (G′) and (H′) show magnifications of the nurse cell oocyte boundary region (white scale bar, 5μm). (I–L) Cytoplasmic movements in stage 9 oocytes visualized by merging ten successive images taken at 7 s intervals. The green channel visualizes autofluorescent yolk granules and GFP, and the red particles represent unidentified organelles that reflect 568 nm light. (I) A wild-type oocyte showing slow ooplasmic seething, which results in slight blurring of the particles. (J) A *capu^G7^/Df(2L)ed^SZ1^* oocyte showing premature fast-directional streaming, which causes the particles to appear as lines in the merged image. (K and L) *capu^G7^/Df(2L)ed^SZ1^* oocyte expressing GFP-Capu (K) or GFP-CapuΔN (L). (M–R′) Stage 9 egg chambers showing MTs visualized by staining with α-Tubulin (M, O, and Q) and *osk* mRNA in situ hybridizations (N, P, and R). (N), (P), and (R) show the fluorescent in situ signal superimposed on a DIC image of the oocyte, whereas (N′), (P′), and (R′) show the fluorescent signal alone. (M, N, N′) Wild-type oocyte; (O, P, P′) *capu^G7^/Df(2L)ed^SZ1^* oocyte; (Q, R, R′) *capu^G7^/Df(2L)ed^SZ1^; GFP-CapuΔN* oocyte.

The posterior recruitment of PAR-1 is mediated by its C-terminal linker domain (Doerflinger et al., 2006). To examine whether the Capu pathway is required for the polarized recruitment of PAR-1 to the posterior cortex of the oocyte, we expressed a GFP-PAR-1 linker domain fusion in *capu*^RK^ *spire*^RP^ double-mutant females. GFP-PAR-1 always formed the same broad posterior crescent in mutant oocytes as in wild-type, indicating that the Capu pathway is not required to form the actin structure that mediates PAR-1 localization to the posterior cortex (Figures 1A and 1B). In addition, the MT cytoskeleton still showed polarity in *capu*^RK^ *spire*^RP^ double-mutant oocytes even though the MTs are aligned around the cortex, since the cortical arrays usually did not extend around the posterior pole (Figures 1C and 1D). This suggests that the posterior crescent of PAR-1 still represses the anchoring or nucleation of MTs at the posterior cortex in the double mutant, as it does in wild-type (Shulman et al., 2000). Although *osk* mRNA localization to the oocyte posterior at stage 9 is abolished in *spire* mutants, a small amount of RNA was often correctly localized to the posterior of *spire* null mutant oocytes at late stages, but not in *capu* or *capu spire* double mutants (see Figure S1 in the Supplemental Data available with this article online; data not shown). Thus, the posterior actin cortex can still anchor *oskar* mRNA that is localized by the facilitated diffusion and trapping mechanism that has been described in late oocytes (Glotzer et al., 1997). These results suggest that the cortical polarity is correctly established in *capu* and *spire* mutants and that Capu and Spire function downstream of this polarity to organize the MT gradient.

### The Rho-Binding Domain of Capu Is Not Required for MT Organization

Other formin-related proteins, such as Bni1p and mDia, are activated by the binding of Rho-GTP to an N-terminal Rho-binding domain (RBD), which relieves autoinhibition by a C-terminal diaphanous autoregulatory domain (DAD) (Alberts, 2001; Evangelista et al., 1997; Otomo et al., 2005). Capu may also be regulated by Rho, because a domain near its N terminus (aa 125–250) binds RhoA in a GTP-dependent manner (Rosales-Nieves et al., 2006). Furthermore, *RhoA* +/+ *capu* double heterozygotes or depletion of RhoA from the oocyte causes MT bundling and premature streaming phenotypes that closely resemble those of *capu* mutants (Rosales-Nieves et al., 2006).

To address whether regulation of Capu by Rho-GTP is required for the polarized organization of the MTs in the oocyte during mid-oogenesis, we generated UASp constructs for both full-length Capu and an N-terminally truncated form of the protein lacking amino acids 1–270 (CapuΔN) as fusion proteins with GFP at their N termini (Figure 1E). We expressed the full-length fusion protein in the female germline using nos-Gal4-VP16 and visualized its localization in living egg chambers (Van Doren et al., 1998). This revealed that Capu is mainly cytoplasmic in both wild-type and *capu* mutant (*capu*^G7^*/Df(2L)ed*^SZ1^) egg chambers, with a weak enrichment at the cortex between germline cells (Figures 1G and 1K, data not shown). The Gal4 system gives mosaic expression in the nurse cells, and some cells therefore express higher levels of the fusion protein than others. Nevertheless, GFP-Capu shows a fairly uniform distribution within the cytoplasm of both the nurse cells and the oocyte, with a slight decrease in levels near the oocyte posterior, probably because of the higher concentration of yolk granules in this region. GFP-CapuΔN shows a very similar distribution to full-length protein, but is not enriched at the cortex between germline cells, suggesting that the cortical localization is mediated by the N-terminal domain (Figures 1H and 1L). We have not been able to obtain an α-Capu antibody to compare the expression levels of the fusion proteins to the level of endogenous Capu. However, western blots with anti-GFP show that GFP-Capu and GFP-CapuΔN are expressed at similar levels (Figure 1F).

Full-length Capu protein suppressed premature streaming and restored normal MT organization in *capu* mutant egg chambers (Figures 1I–1K, Tables S1 and S2). The GFP-CapuΔN transgene also completely suppressed premature cytoplasmic streaming in *capu* oocytes and rescued the MT organization, as visualized by α-Tubulin staining (Figures 1L and 1M–1Q). As an indirect marker of the MT polarization, we examined the localization of *osk* mRNA to the posterior of the oocyte. *osk* mRNA formed a posterior crescent in all *capu*; *GFP-CapuΔN* stage 9–10A oocytes, indicating that the polarity of the MT cytoskeleton was normal (Figures 1N–1R, Table S3). The N-terminal Rho-binding domain of Capu is therefore dispensable for correct MT polarization, arguing against models in which the spatial control of Capu through this domain establishes a polarized cue that organizes the MTs. Furthermore, these experiments show that cortical enrichment of Capu is not required for normal MT organization.

### GFP-CapuΔN Rescues *spire* Mutants at Stage 9

**Figure 2:**
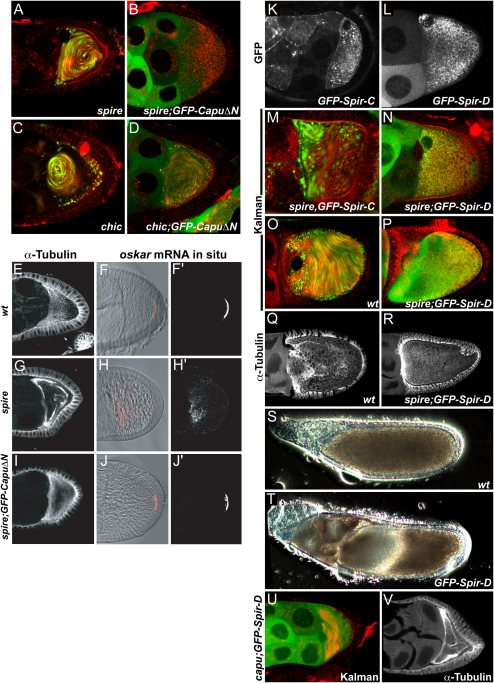
Rescue of *spire* by GFP-CapuΔN and GFP-Spir-D (A–D) Cytoplasmic movement at stage 9 in *spire*^RP^ (A) *spire*^RP^; *GFP-CapuΔN* (B), *chic*^1320^*/chic*^221^ (C), and *chic*^1320^*/chic*^221^; *GFP-CapuΔN* oocytes (D). (E–J′) MT staining (E, G, and I) and *osk* mRNA localization (F, F′, H, H′, J, and J′) in wild-type (E, F, and F′), *spire*^RP^ (G, H, and H′), and *spire*^RP^; *GFP-CapuΔN* (I, J, and J′) oocytes at stage 9. (K) GFP-Spir-C localization at stage 9. (L) GFP-Spir-D localization at stage 9. (M and N) Cytoplasmic movements in stage 9 *spire*^RP^/*Df(2L)Exel*^6046^ oocytes expressing GFP-Spir-C (M) or GFP-Spir-D (N). (O and P) Cytoplasmic movements at stage 11 in wild-type (O) and in a *spire*^RP^/*Df(2L)Exel*^6046^ oocyte expressing GFP-Spir-D (P). (Q and R) α-Tubulin stainings of stage 11 wild-type (Q) and *spire*^RP^/*spire*^2F^; *GFP-Spir-D* egg chambers (R). (S and T) Phase-contrast images of stage 12 egg chambers, showing the distribution of yolk in wild-type (S) and *spire*^RP^/*Df(2L)Exel*^6046^; *GFP-Spir-D* oocytes (T). (U–V) *capu*^G7^*/Df(2L)ed*^SZ1^ egg chambers expressing GFP-Spir-D, showing premature ooplasmic streaming at stage 9 (K) and premature formation of cortical MT arrays (L).

To analyze whether the Capu constructs were able to suppress premature streaming in the absence of Profilin or Spire, we expressed the Capu transgenes in *spire* and *chic* mutants. Expression of GFP-CapuΔN in females homozygous for a null allele of *spire, spire^RP^*, blocked premature streaming until the end of stage 9 (Figures 2A and 2B, Table S1). Expression of the full-length GFP-Capu also suppressed premature streaming at stage 9 (Table S1). In contrast, neither GFP-Capu nor GFP-CapuΔN expression suppressed premature streaming in *chic^1320^/chic^221^* oocytes (Figures 2C and 2D, Table S1).

To characterize the rescue of *spire* by GFP-CapuΔN in more detail, we examined the MT organization and *osk* mRNA localization (Figures 2E–2J′). In *spire* mutant egg chambers, the MTs are aligned in arrays along the oocyte cortex at stage 9, instead of the wild-type anterior-to-posterior gradient, and *osk* mRNA fails to localize to the posterior cortex (Figures 2G–2H′). Expression of GFP-CapuΔN in these egg chambers produced a nearly wild-type gradient of MTs in the oocyte at stage 9 (Figure 2I, Table S2). This rescue is only temporary, however, because most oocytes showed cortical arrays of MTs at stage 10A. *osk* mRNA localization was also partially rescued, with more than half of the oocytes showing a normal posterior crescent (Figures 2J and 2J′, Table S3). The MT organization and *oskar* mRNA localization in *spire* mutant egg chambers expressing GFP-Capu were also partially rescued, but with lower penetrance, suggesting that GFP-CapuΔN is more active.

Since *spire* mutants cause a fully penetrant phenotype in the presence of endogenous Capu, the GFP-Capu fusion proteins must be more active than the endogenous protein, either because they are overexpressed and/or because the N-terminal GFP fusion activates Capu. To investigate the latter possibility, we created a UASp-Capu transgene that drives the expression of full-length untagged Capu. Expression of this construct also partially rescued *spire*^RP^ mutants, although less effectively than the GFP-tagged proteins (Table S2). Thus, overexpression of wild-type Cappuccino can partially compensate for the absence of Spire. Since increased Capu activity can rescue both the MT and *osk* mRNA localization defects of *spire* mutants, Spire is not essential for the polarization of the oocyte MT cytoskeleton, ruling out models in which the spatial control of Spire plays a role in establishing asymmetry.

### Spire D Is Required for MT Organization in the Oocyte

We also investigated the role of Spire by expressing GFP-Spir-C and GFP-Spir-D constructs in the female germline under the control of nos-Gal4-VP16 (Rosales-Nieves et al., 2006). GFP-Spir-D shows a punctate distribution throughout the cytoplasm of the living oocytes, suggesting that it is associated with small organelles or vesicles, but shows no obvious enrichment at the cortex, while GFP-Spir-C shows a vesicular and cortical distribution as previously described (Rosales-Nieves et al., 2006) (Figures 2K and 2L).

Expression of GFP-Spir-C did not rescue the premature cytoplasmic streaming of *spire* mutant oocytes (Figure 2M). By contrast, expression of GFP-Spir-D suppressed premature streaming in *spire* null mutants completely (Figure 2N, Table S1). Furthermore, all *spire*; *GFP-Spir-D* oocytes formed a normal anterior-to-posterior gradient of MT, and *osk* mRNA always localized to the posterior pole (Tables S2 and S3). Thus, Spire D rescues all of the phenotypes of *spire* null mutants at stages 9–10 of oogenesis, whereas Spire C appears to be dispensable. These results argue against the model in which Spire D is a negative regulator that inhibits the anchoring of microtubules to the actin cortex by Spire C and Capu. Spire D does not interact with MT in vitro and contains the repeated WH2 domains that nucleate actin filaments in vitro (Quinlan et al., 2005; Rosales-Nieves et al., 2006). This suggests that the main function of Spire in the oocyte is related to the formation of F-actin structures rather than MT anchoring.

Surprisingly, expression of GFP-Spir-D also prevented the fast streaming of the oocyte cytoplasm that normally occurs during stages 11–13 (Figures 2O and 2P). The MTs failed to align into subcortical arrays during these stages and remained in the anterior-posterior gradient that is normally seen during stages 9–10A (Figures 2Q and 2R). As a consequence of the lack of streaming, the contents of the oocyte were not mixed during nurse cell “dumping,” and the oocytes adopted a stratified appearance, with the yolk at the posterior and the clear cytoplasm from the nurse cells at the anterior (Figures 2S and 2T). This activity of GFP-Spir-D still requires endogenous Capu, since it did not rescue the premature streaming and MT phenotypes of *capu* mutants (Figures 2U and 2V).

### Capu and Spire Are Required for the Formation of an Actin Mesh in the Oocyte Cytoplasm

**Figure 3:**
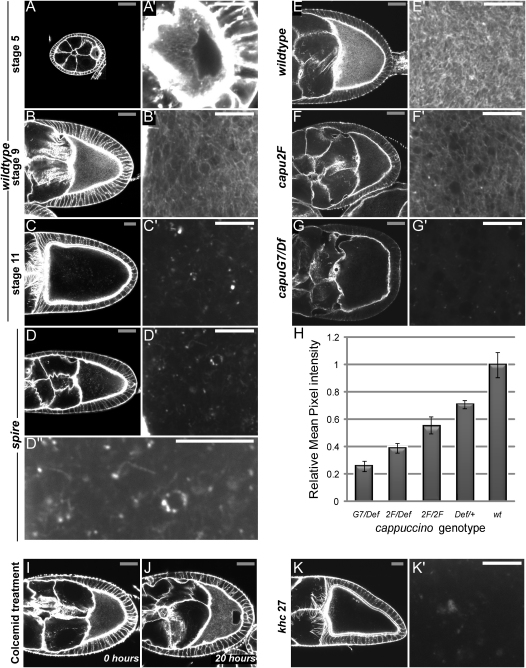
Capu and Spire Are Required for the Formation of an Actin Mesh in the Oocyte Cytoplasm Egg chambers stained with TRITC-Phalloidin to label F-actin (gray scale bars, 30 μm; white scale bars, 10 μm). (A and A′) A stage 5 wild-type egg chamber. (A′) Magnification of the oocyte showing the actin mesh in the oocyte cytoplasm. (B and B′) A stage 9 wild-type egg chamber. (C and C′) A stage 11 wild-type egg chamber. (D, D′, and D″) A stage 9 *spire*^RP^ egg chamber. (E–G) Wild-type (E), *capu*^2F^ (F), *capu*^G7^*/Df(2L)ed^SZ^*^1^ (G) stage 9 egg chambers processed in parallel, with Phalloidin staining after fixation. (H) Bar diagram showing the relative average of mean pixel intensities of the cytoplasmic F-actin staining in stage 9 egg chambers of various genotypes (*capu*^G7^*/Df(2L)ed^SZ^*^1^, *capu*^2F^*/Df(2L)ed*^SZ1^, *capu*^2F^, *Df(2L)ed*^SZ1^/+, wild-type). The vertical lines show the standard deviations in mean pixel intensities. (I and J) Wild-type egg chambers before (I) and after a 20 hr treatment with the MT-depolymerizing drug colcemid (J). (K and K′) A *khc^27^* germline clone.

Although the results above suggest that the Capu pathway functions through nucleation of actin filaments in the oocyte, previous work has shown that this pathway is not required for the formation of the actin cortex or the ring canals, which are the only populations of F-actin that can be detected by normal staining procedures (Magie et al., 1999). Furthermore, it seems likely that Capu pathway organizes a dynamic population of actin, since the premature streaming phenotype of *capu* class mutants can be phenocopied by a few minutes' exposure to actin-depolymerizing drugs (Manseau et al., 1996). We therefore decided to reexamine the organization of F-actin in the oocyte using a rapid-fixation procedure in which Phalloidin was added to the fixative to preserve unstable actin structures (Frydman and Spradling, 2001). This revealed a uniform network of actin filaments throughout the cytoplasm of the oocyte that is not present in the nurse cells. This ooplasmic actin mesh can be detected from stage 5 of oogenesis and persists during mid-oogenesis when the polarized MT cytoskeleton is required for localization of the anterior and posterior determinants (Figures 3A and 3B). The actin mesh starts to disappear from the center of the oocyte during stage 10B, coincident with the initiation of fast ooplasmic streaming, and is absent at stage 11, when F-actin is visible in a few dense dot-like structures (Figures 3C and 3C′).

**Figure 4:**
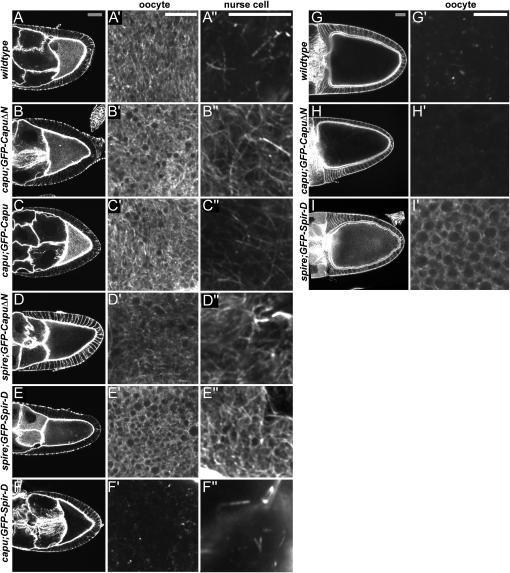
The Effects of Capu and Spire Constructs on Actin Mesh Formation Stage 9 (A–F) and stage 11 (G–I) egg chambers stained with TRITC-Phalloidin to label F-actin (gray scale bars, 30 μm; white scale bars, 10 μm). (A′)–(I′) show magnifications of the oocyte cytoplasm, imaged 10 μm (A′–G′) or 20 μm from the cortex (H′–J′), while (A″)–(G″) show equivalent regions of the nurse cell cytoplasm. (A) Wild-type, (B) *capu*^G7^*/Df(2L)ed*^SZ1^; *GFP-CapuΔN*, (C) *capu*^G7^*/Df(2L)ed*^SZ1^; *GFP-Capu*, (D) *spire*^RP^; *GFP-CapuΔN*, (E) *spire*^RP^/*spire*^2F^; *GFP-Spir-D*, (F) *capu*^G7^*/Df(2L)ed*^SZ1^; *GFP-Spir-D*, (G) wild-type, (H) *capu*^G7^*/Df(2L)ed^SZ1^*; *GFP-CapuΔN*, (I) *spire*^RP^/*Df(2L)Exel*^6046^; *GFP-Spir-D*.

The actin mesh in the oocyte is not formed in strong *capu*, *spire*, and *chic* mutants, and instead any remaining cytoplasmic F-actin forms small dots (Figures 3D–3G, Figure S2). The loss of the actin mesh in Capu pathway mutants suggests that these three actin regulators act together to assemble cytoplasmic actin filaments, and demonstrates that the mesh observed in wild-type is not an artifact of Phalloidin-induced polymerization during the fixation. Indeed, the visualization of the actin mesh does not depend on the presence of Phalloidin in the fixative, since an identical mesh is observed when Phalloidin is added after rapid fixation (Figures 3E–3G, 4A–4C, 4F–4H, and 4J; Figures S2 and S3).

There is a good correlation between the penetrance of the premature streaming and ventralized egg phenotypes of mutants in *capu*, *spire*, and *chic* and the strength of their effect on the formation of the actin mesh. For example, only two-thirds of *chic^1320^* homozygotes (16/25) show premature streaming at stages 8–9, and these egg chambers still retain an actin mesh-like structure in the oocyte cytoplasm, although this is much less dense than the wild-type actin mesh (Figures S2A–S2D). Quantification of the mean pixel intensity of the cytoplasmic TRITC-Phalloidin signal in stage 9 oocytes that had been stained and imaged under identical conditions revealed that the amount of F-actin decreased in proportion with the reduction in Profilin activity (Figure S2). Another example is *capu^2F^*, which is a point mutation in the FH2 domain and one of the weakest alleles of *capu*, with only 5% of the eggs laid by homozygotes showing a ventralized phenotype (Emmons et al., 1995). Consistent with this, there is a residual actin mesh in *capu^2F^* oocytes, although it is always much weaker than in wild-type (Figures 3E–3G). Quantification of mean pixel intensity again showed that the level of F-actin staining correlated with the amount of Capu activity, with *w* > +/*Df(2L)ed^SZ1^ > capu^2F^/capu^2F^ > capu^2F^/Df(2L)ed^SZ1^ > capu^G7^/Df(2L)ed^SZ1^* (Figure 3H, Figure S2). Since the actin mesh is weaker in *capu* or *chic* heterozygotes, the endogenous levels of both proteins appear to be limiting for actin mesh formation.

### Actin Mesh Formation Is Independent of MTs

When the actin mesh is present, the oocyte MTs always form an anterior-posterior gradient, whereas they form cortical arrays when the mesh is absent, as in Capu pathway mutants or in late oocytes. This correlation raises the question of whether the Capu pathway is required for the assembly of the actin mesh, which in turn organizes the MT gradient, or whether the pathway first organizes the MTs, which then control the formation of the actin mesh. To distinguish between these possibilities, we asked whether the MT cytoskeleton plays any role in the formation of the actin mesh by examining egg chambers from females fed colcemid for 20 hr, which rapidly disassembles all MTs in the oocyte. As expected, colcemid treatment blocks the MT-dependent migration of the oocyte nucleus, which remains at the posterior cortex. The actin mesh is still present, however, indicating that it is MT independent (Figures 3I and 3J).

The disappearance of the actin mesh during stage 10B coincides with the onset of fast cytoplasmic streaming, raising the possibility that the mesh is disassembled by the vigorous cytoplasmic flows during this period. Since streaming is driven by the plus-end-directed MT motor protein, Kinesin, this can be tested by examining the mesh in a null mutant of *kinesin heavy chain*, *khc^27^*, which blocks all cytoplasmic flows in the oocyte (Palacios and St Johnston, 2002). The actin mesh still disappears on schedule in *khc*^27^ germline clones, indicating that its disassembly is controlled by some other mechanism (Figure 3K). Thus, actin mesh formation is independent of MT and Kinesin, strongly suggesting that the Capu pathway directly regulates its assembly.

### GFP-CapuΔN and GFP-Spir-D Induce an Ectopic Actin Mesh in the Nurse Cells

Next, we examined whether the Capu transgenes induced actin mesh formation in *capu* and *spire* mutants. GFP-CapuΔN expression in *capu* egg chambers rescued the actin mesh in the oocyte up to stage 10B and also induced the formation of an ectopic actin mesh-like structure in the nurse cells (Figures 4A and 4B). The actin mesh in the oocyte appeared denser than in wild-type, whereas the ectopic mesh in the nurse cells was generally fainter, with a density that corresponded to the level of GFP-CapuΔN expression in each cell. Furthermore, GFP-CapuΔN expression also induced an actin mesh in both the oocyte and nurse cells of *spire* mutants at stage 9, consistent with its ability to rescue their MT and *osk* mRNA localization phenotypes, although this mesh appeared less dense than in wild-type (Figure 4D). GFP-CapuΔN did not induce the formation of an actin mesh in *chic* mutant egg chambers, in line with its failure to suppress premature streaming in these oocytes (Figure S3). We also tested whether wild-type GFP-Capu or untagged Capu induced formation of the cytoplasmic F-actin network in *capu* and *spire* mutant oocytes and observed that both constructs completely rescued mesh formation in *capu*, but only weakly rescued *spire* (Figure 4C; data not shown).

GFP-Spir-D expression rescued the actin mesh in *spire* mutant oocytes and induced the formation of an ectopic actin mesh in the nurse cells that was significantly denser than in GFP-CapuΔN-expressing egg chambers, and correlated with the level of Spire D overexpression (Figure 4E). Expression of GFP-Spir-D did not lead to formation of an actin mesh in either the oocyte or the nurse cells of *capu* mutants, however, indicating that endogenous Capu is required for the induction of the actin mesh by Spire D (Figure 4F).

None of the Capu constructs affected the disappearance of the actin mesh during stage 10B (Figures 4G and 4H). However, the actin mesh in the oocyte was not disassembled during stages 10B–11 in egg chambers expressing GFP-Spir-D, and persisted throughout the rest of oogenesis (Figure 4I). This may explain why GFP-Spir-D expression prevents the normal late cytoplasmic streaming in the oocyte during stages 10B–13.

### CapuΔN Stabilizes the Actin Mesh and Inhibits Latrunculin-A-Induced Premature Streaming

**Figure 5:**
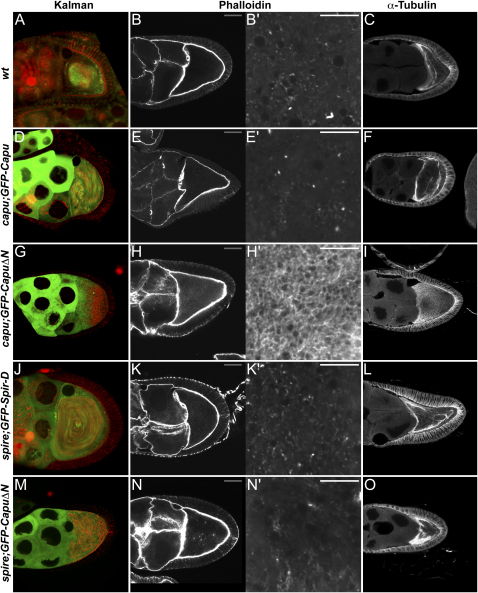
CapuΔN Stabilizes the Actin Mesh against Latrunculin-A-Induced Depolymerization Confocal images of stage 9 egg chambers after treatment with Latrunculin A. (A), (D), (G), (J), and (M) show the cytoplasmic movements in living egg chambers; (B), (E), (H), (K), and (N) are egg chambers stained with TRITC-Phalloidin to label F-actin (gray scale bars, 30 μm); (B′), (E′), (H′), (K′), and (N′) show magnifications of the F-actin staining in the oocyte cytoplasm imaged 10 μm from the cortex (white scale bars, 10μm); (C), (F), (I), (L), and (O) show MTs visualized by α-Tubulin staining. (A–C) Wild-type oocytes; (D–F) *capu*^G7^*/Df(2L)ed*^SZ1^; *GFP-Capu*; (G–I) *capu*^G7^*/Df(2L)ed*^SZ1^; *GFP-CapuΔN*; (J–L) *spire*^2F^*/spire*^RP^; *GFP-Spir-D*; (M-O) *spire*^2F^*/spire*^RP^; *GFP-CapuΔN*.

Treatment of oocytes with drugs that sequester G-actin induces premature ooplasmic streaming within a few minutes (Manseau et al., 1996). This suggests that the Capu- and Spire-dependent actin mesh is an unstable structure with a high turnover. To test this, we fed Latrunculin A to wild-type females and *capu* females expressing the different Capu constructs and analyzed the induction of premature streaming and the disappearance of the actin mesh in the oocyte. Eight hours of Latrunculin A feeding induced a fully penetrant premature streaming phenotype and the disappearance of the actin mesh (Figures 5A and 5B, Table S4). The same effect was observed in *capu* mutant oocytes expressing GFP-Capu (Figures 5D and 5E). By contrast, Latrunculin A treatment had no effect on the cytoplasmic movements and actin mesh in GFP-CapuΔN-expressing *capu* egg chambers (Figures 5G and 5H). Thus, GFP-CapuΔN stabilizes the actin mesh in the presence of Latrunculin A. These results were confirmed by visualizing the MT cytoskeleton in the treated oocytes. Latrunculin A induced the premature formation of cortical arrays of MTs in 100% of wild-type and nearly 97% of *capu*; *GFP-Capu* egg chambers, whereas all *capu*; *GFP-CapuΔN* oocytes still displayed a wild-type anterior-posterior gradient of MTs (Figures 5C, 5F, and 5I; Table S5).

Expression of GFP-Spir-D was not sufficient to stabilize the actin mesh or suppress the Latrunculin A-induced premature streaming (Figures 5J–5L). We then asked whether Spire is required for the GFP-CapuΔN-induced stabilization of the actin mesh. As in wild-type, 8 hr of Latrunculin A feeding induced loss of the actin mesh in *spire* mutant oocytes expressing GFP-CapuΔN, leading to premature streaming and the formation of cortical MTs (Figures 5M–5O). Thus, the ability of GFP-CapuΔN to stabilize the actin mesh in the presence of Latrunculin A depends on endogenous Spire.

### The Role of the Actin Mesh in MT Organization

The presence of the actin mesh correlates perfectly with the formation of an anterior-posterior gradient of MTs in the oocyte and with the absence of cortical MT bundles and fast cytoplasmic streaming, strongly suggesting that the mesh acts upstream of the MTs and is required for their polarized organization during stages 7–9. Although this could reflect a direct role of the actin mesh in organizing the MT cytoskeleton, it has recently been shown that the anterior-posterior gradient of MTs typical of stage 7–9 oocytes persists during stage 10B/11 in mutants in the kinesin heavy chain (Serbus et al., 2005). Since kinesin is required for fast cytoplasmic streaming, this observation has led to the proposal that the cortical bundles of MTs that form at these late stages are caused by kinesin-dependent cytoplasmic flows, which wash the MTs into alignment around the cortex.

**Figure 6:**
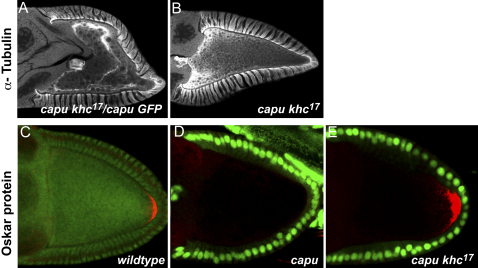
A Slow Mutant in Kinesin Rescues the *capu* Phenotype (A) α-Tubulin staining of a stage 10A *capu*^RK^*FRTG13 nlsGFP/capu*^G7^*FRTG13 khc*^17^ egg chamber. (B) α-Tubulin staining of a stage 10A egg chamber containing a *capu^RK^ FRTG13 khc^17^/capu*^G7^*FRTG13 khc^17^* germline clone. (C) A wild-type stage 9 egg chamber stained for Oskar protein (red), showing its localization at the posterior pole of oocyte. (D) A stage 9 egg chamber containing a *capu*^RK^*FRTG13* +/*capu*^G7^*FRTG13* + germline clone (marked by the absence of green GFP signal in the germline). Oskar protein (red) is not expressed at the posterior of the oocyte in the *capu* null mutant egg chamber. (E) A stage 9 egg chamber containing a *capu*^RK^*FRTG13 khc*^17^*/capu*^G7^*FRTG13 khc*^17^ germline clone. Homozygosity for *khc^17^* rescues the posterior localization of Oskar (red) in the *capu* mutant.

To test whether the actin mesh is directly required for the initial establishment of the MT gradient, or if it only regulates the MT gradient indirectly by constraining kinesin-dependent cytoplasmic flows, we generated germline clones that were doubly mutant for *capu* and *khc*^17^, a missense mutation in the kinesin motor domain that reduces its speed of movement (Serbus et al., 2005). The *khc*^17^ mutant suppressed the premature streaming phenotype of *capu*, as expected, and also rescued the polarized organization of the MT cytoskeleton (Figures 6A and 6B). More importantly, *khc*^17^ also suppressed the polarity defect of the *capu* mutant and restored the formation of a posterior crescent of Osk protein (Figures 6C–6E). The actin mesh is therefore dispensable when the speed of kinesin-dependent movements is reduced. This suggests that the actin mesh maintains the polarized organization of the MT cytoskeleton indirectly by restricting kinesin-dependent cytoplasmic flows. To test this hypothesis, we also made time-lapse movies of the movements of MTs labeled with Tau-GFP in wild-type and *capu* and *spire* mutant oocytes at stage 9. Although the MTs are fairly static in wild-type, they are washed into alignment near the cortex in the mutant oocytes, consistent with the idea that they are reorganized by kinesin-dependent flows (Movies S1 and S2 and data not shown).

## Discussion

Formin-related proteins play a key role in cell polarity in a number of systems and usually show a highly polarized distribution to one end of the cell. For example, Bni1p and For3p localize to the poles of budding and fission yeast, respectively, where they nucleate actin cables that are required for polarized growth, while mDia stabilizes MT plus ends at the leading edge of migrating fibroblasts (Evangelista et al., 2002; Martin et al., 2005; Palazzo et al., 2001). Although the *Drosophila* forming-related protein, Capu, is similarly required for the polarization of the oocyte MT cytoskeleton and for the formation of both the anterior-posterior and dorsal-ventral axes, our results demonstrate that it regulates MTs by a very different mechanism from these other formins. Neither Capu nor its partner Spire shows a polarized distribution within the oocyte, nor do they play a direct role in MT organization in a particular region of the cell. Instead, they act together with Profilin to assemble an isotropic actin mesh in the oocyte cytoplasm, which maintains the polarized arrangement of MTs by suppressing kinesin-dependent cytoplasmic streaming.

This function for Capu and Spire contrasts with the recent proposal that they act at the oocyte cortex to regulate cortical polarity and to crosslink the actin and MT cytoskeletons (Rosales-Nieves et al., 2006). Our results argue against this model for several reasons. First, cortical polarity appears to be unaffected in *capu* and *spire* mutant egg chambers. PAR-1 still localizes normally to the posterior cortex, and *osk* mRNA is specifically anchored at the posterior in *spire* mutant egg chambers, indicating that this region of the cortex is different from the anterior and lateral domains. Furthermore, the MTs show a normal association with the anterior and lateral cortex in *capu* and *spire* mutants, as is most clearly demonstrated by the wild-type MT arrangement in *capu* mutants in which kinesin function is impaired.

Second, although Capu and Spire interact with MTs in vitro, this activity does not appear to be required for their function in vivo. Spire D, which lacks the MT-binding domain, completely suppresses cytoplasmic streaming at all stages, whereas Spire C, which contains the domain, has no effect on the *spire* mutant phenotype. Thus, the MT-binding activity of Spire is not required for its in vivo activity. A similar argument can made for the MT-binding activity of Capu. Capu binds MT in vitro through its FH2 domain, and a P597T substitution in the *capu^2F^* allele blocks this activity (Rosales-Nieves et al., 2006). Despite this loss of MT binding, *capu^2F^* has the weakest phenotype of all *capu* alleles examined, indicating that the inability to interact with MT has little effect on Capu's in vivo activity. Furthermore, the weak phenotype of *capu^2F^* is more easily explained by an effect on actin nucleation, since we observe a clear reduction in the actin mesh in *capu^2F^* homozygous oocytes, although the P597T mutation was reported to have minimal effect on actin nucleation in vitro.

The localization of Capu and Spire also argues against a model in which they act exclusively to anchor MTs to the cortex. Neither GFP-tagged Capu nor Spire D is enriched at the oocyte cortex when visualized in living oocytes, even though these fusion proteins are functional, since they rescue the strongest alleles of *capu* and *spire*, respectively. This contrasts with the previous study in which both proteins were reported to localize to the oocyte cortex, and may reflect the fact that the latter examined their distribution in detergent-extracted and fixed samples (Rosales-Nieves et al., 2006). It therefore seems unlikely that the direct crosslinking of actin and MTs by Capu or Spire at the cortex plays a significant role in their function in the oocyte.

Instead, our results indicate that the Capu pathway functions to organize a dynamic network of actin filaments throughout the oocyte cytoplasm. This actin mesh is lost in *capu*, *spire*, and *chic* mutants, indicating that Capu, Spire, and Profilin are necessary for its formation. Furthermore, overexpression of Capu or Spire D induces an ectopic mesh in the nurse cells, while Spire D induces an ectopic mesh in late oocytes, strongly suggesting that both proteins play a direct role in its assembly. Indeed, the role of Capu in the formation of the cytoplasmic actin mesh may explain the seemingly paradoxical observation that *capu* mutants cause an increase in the amount of cortical actin in the oocyte (Magie et al., 1999; Rosales-Nieves et al., 2006). The failure to form the actin mesh in *capu* mutants should lead to a rise in the concentration of free G-actin in the oocyte, which may promote excess actin polymerization at the oocyte cortex by a Capu- and Spire-independent mechanism.

The presence of the ooplasmic actin mesh correlates perfectly with the polarized arrangement of the MTs in the oocyte. The mesh is present from stage 5 to stage 10A of oogenesis, which is the period during which the anterior-posterior gradient of MT persists, and the disappearance of the mesh at stage 10B coincides with the onset of fast cytoplasmic streaming and the rearrangement of the MT into parallel cortical arrays. Furthermore, the loss of the mesh in *capu*, *spire*, and *chic* mutants leads to premature streaming and the precocious formation of cortical MT arrays, whereas the overexpression of Spire D maintains the mesh during stage 11 and suppresses the normal rearrangement of the MT and streaming at this stage. Indeed, the density of the mesh correlates with the severity of the mutant phenotype, since the weakest alleles of *capu* and *chic* cause a reduction in the mesh without abolishing it entirely.

This revised view of the function of Capu and Spire is consistent with the known biochemical properties of the other formin-related proteins and Spire. In vitro studies have shown that formin-related proteins nucleate actin filaments through their FH2 domains and then remain associated with the barbed end, which they protect from actin-capping proteins, while increasing the rate of elongation through the interaction of the FH1 domain with Profilin/Actin complexes (Goode and Eck, 2007). Although Capu is not a typical formin, it contains well-conserved FH1 and FH2 domains, nucleates actin in vitro, and has been shown to interact with Profilin in yeast two-hybrid assays (Emmons et al., 1995; Manseau et al., 1996; Quinlan et al., 2005). Furthermore, the protection of the actin mesh from Latrunculin A-induced depolymerization by CapuΔN is consistent with a model in which the protein remains associated with the barbed ends and prevents their disassembly. Spire, on the other hand, nucleates actin filaments from their pointed ends and caps this end of the filament as it grows (Quinlan et al., 2005). Thus, both Capu and Spire have the capacity to nucleate and stabilize actin filaments, raising the possibility that each protein independently nucleates and stabilizes actin filaments in the mesh. This is consistent with the observation that overexpression of Capu can induce the formation of an actin mesh in the absence of Spire. The mesh induced by overexpressed Capu alone is significantly weaker than normal, however, and persists for a shorter time, while Spire D cannot form a mesh in the absence of Capu. Furthermore, the ability of GFP-CapuΔN to stabilize the actin mesh in the presence of Latrunculin depends on endogenous Spire activity. Thus, the two proteins must cooperate to form a normal mesh, and one possibility is that they assist each other by capping the opposite ends of filaments nucleated by the other. Since Spire D associates with Capu in vitro, it is even possible that they collaborate to nucleate the same filament and remain attached to opposite ends as it grows.

Although the mesh is essential for the polarized arrangement of the MTs in the oocyte, it appears to play a permissive rather than an instructive role, because the defects in MT organization and *osk* mRNA localization caused by its loss can be rescued by slowing the speed of kinesin. This suggests that the mesh normally serves to restrain kinesin-dependent motility and that the rearrangement of the MTs and premature streaming are a consequence of unrestricted kinesin activity. Kinesin is required both for the slow disorganized cytoplasmic movements during stage 9, called seething, and for the rapid directional streaming at stage 11, leading to the proposal that the motor generates ooplasmic flows by moving large organelles or vesicles along MTs (Palacios and St Johnston, 2002; Serbus et al., 2005). This suggests the following model for how loss of the actin mesh and unrestrained kinesin motility cause the rearrangement of the MT. In the absence of the actin mesh, there is an increase in the speed or frequency of kinesin-dependent organelle transport, resulting in a concomitant increase in the strength of the cytoplasmic flows that these movements generate. Since the MTs move with the cytoplasmic flows (see Movie S2), the stronger flows will start to wash the MTs into alignment, thereby aligning the kinesin-dependent organelle movements, which will amplify the cytoplasmic flows still further. This positive-feedback loop then continues to coordinate and increase the flows until all MTs have been washed to the oocyte cortex, with the oocyte cytoplasm rapidly rotating inside.

This model raises the question of how the actin mesh restrains the kinesin-dependent cytoplasmic flows to prevent their amplification into cytoplasmic streaming. This could be an entirely passive process, in which the actin mesh increases the viscosity of the oocyte cytoplasm, thereby increasing the drag on kinesin-dependent transport. However, we favor a model in which the mesh plays a more direct role in the inhibition of kinesin-mediated movement of the cytoplasm, and one attractive possibility is that it tethers the cargoes of kinesin that generate the flows, thereby limiting their movement. One way that the organelles might be tethered to the actin mesh is by binding to either Capu or Spire, and it is interesting to note that Spire-D shows a punctate distribution that is consistent with an association with a population of organelles or vesicles. In addition, full-length Spire contains an mFYVE domain that is predicted to target it to endosomal membranes, and has been shown to colocalize with Rab11 on vesicular structures when expressed in tissue culture cells (Kerkhoff et al., 2001; Otto et al., 2000). This tethering mechanism is very similar to the function of mDia in the anchoring of endosomes to actin at the cell periphery, which inhibits their movement along MT, and also resembles the tethering of mitochondria in neuronal cells, where mDia nucleates actin filaments that anchor the mitochondria, without affecting the motility of lysosomes or peroxisomes (Fernandez-Borja et al., 2005; Gasman et al., 2003; Minin et al., 2006).

A third possibility is that the mesh anchors the MTs within the cytoplasm and prevents them from being washed into alignment at the cortex by the cytoplasmic flows. As discussed above, it seems unlikely that direct crosslinking of actin and MT by Capu and Spire is important in vivo, but some other protein may anchor the MT to actin. Alternatively, the actin and MTs could be crosslinked indirectly. For example, Capu or Spire could interact with a vesicle or organelle that is associated with MT, thereby linking the two cytoskeletons.

The formation of the actin mesh must be tightly regulated both spatially and temporally, since the mesh normally forms only in the oocyte and not the nurse cells and is disassembled during stage 10B to allow the onset of rapid streaming. Both Capu and Spire bind Rho-GTP, raising the possibility that one or both proteins are regulated by Rho (Rosales-Nieves et al., 2006; Wellington et al., 1999). Indeed, we generated the GFP-CapuΔN construct to test if deletion of its Rho-binding domain would lead to a constitutively active form of the protein. However, overexpression of GFP-Capu or of full-length untagged Capu produces very similar effects to GFP-CapuΔN. The only obvious difference between the three constructs is the ability of CapuΔN to protect the actin mesh from Latrunculin A-induced depolymerization, but it is unclear whether this is due to constitutive activation of Capu or some other alteration to its activity. More importantly, our data suggest that the regulation of Capu activity is unlikely to determine where and when the mesh forms. Although overexpressed Capu can assemble a mesh in both the oocyte and the nurse cells in the absence of Spire until stage 10A, Spire D cannot induce the formation of an actin mesh in the absence of Capu. The ability of Spire to form an ectopic mesh in the nurse cells and in late oocytes therefore implies that endogenous Capu must be active in the nurse cells and during the late stages of oogenesis. This suggests that the regulation of Spire determines the temporal and spatial control of actin mesh formation and disassembly.

In summary, our results suggest that the Capu pathway controls the formation of an actin mesh, which acts as a switch between two alternative states of the oocyte cytoplasm, both of which are essential for the formation of a viable egg. During stages 5–10A, the mesh inhibits kinesin-dependent motility to allow the formation of the anterior-posterior MT array that directs the localization of *oskar* and *gurken* mRNAs, and this establishes the polarity of both body axes. Once *oskar* mRNA has been localized and anchored to the oocyte cortex and Gurken has signaled to polarize the dorsal-ventral axis, the actin mesh is disassembled. This relieves the inhibition of kinesin-dependent organelle movement and switches on fast ooplasmic streaming. As a result, the oocyte cytoplasm becomes thoroughly mixed with the cytoplasm that enters from the nurse cells during nurse-cell dumping, leading to a uniform distribution of maternal proteins and mRNAs throughout the egg. This is important for subsequent development, because most housekeeping functions in the embryo depend on maternal gene products, which must be evenly distributed in the egg, so that they are equally partitioned into all cells.

## Experimental Procedures

### Molecular Biology

The GFP-Capu transgenes were generated by inserting either the entire coding region of *capu* (CG3399-RA) or a region corresponding to amino acids 271–1059 into pUASpPL-GFP to create pUASpPL-GFP-Capu and pUASpPL-GFP-CapuΔN. Untagged full-length Capu was made by placing the entire *capu* coding region into the pUASpPL expression vector.

### *Drosophila* Stocks

We used the following transgenes and mutant stocks in this study: UASpGFP-Par1-linker domain (Doerflinger et al., 2006): nos-Gal4-VP16 (Van Doren et al., 1998); UASp-GFP-Spir-D and UASp-GFP-Spir-C (Rosales-Nieves et al., 2006); capu^RK^, capu^G7^ (Manseau and Schupbach, 1989); capu^2F^(Emmons et al., 1995); Df(2L)ed^SZ1^ (Presgraves, 2003); spire^RP^ (Manseau and Schupbach, 1989); spire^2F^ (Wellington et al., 1999); chic^1320^(Cooley et al., 1992); chic^221^ (Verheyen and Cooley, 1994), khc^17^, and khc^27^(Brendza et al., 1999). capu khc^17^ germline clones were generated by heat-shocking hs-FLP/+; capu^RK^ FRTG13 nlsGFP/ capu^G7^ FRTG13 khc^17^ larvae for 2 hr on 3 consecutive days.

### Microscopy and Staining

The localization of GFP-fusion proteins and cytoplasmic movements were examined in living oocytes using a BioRad 1024 Inverted Confocal Microscope. Cytoplasmic movements were analyzed by collecting 10 consecutive scans using the Kalman algorithm as described by Palacios and St Johnston (2002). In situ hybridizations and antibody stainings were performed according to standard protocols. Antisense *oskar* RNA probes for in situ hybridizations were synthesized using the DIG RNA Labeling mix (Roche). Cy3-conjugated IgG mouse anti-DIG antibody (Jackson ImmunoResearch Laboratories) was used at 1:400, FITC-conjugated Mouse anti-Tubulin Antibody (Sigma) was used at a concentration of 1:250, and Rabbit anti-Oskar (gift from A. Ephrussi) was used at 1:500.

### Visualization of the Actin Mesh

To visualize dynamic actin structures in the oocyte, we dissected egg chambers directly into a solution of 10% formaldehyde, 1X PBS, and 0.01% Tween-20 and fixed them for 30 min. This was done in either fixative containing the actin-stabilizing drug Phalloidin at a concentration of 12.5 μg/ml TRITC-Phalloidin (Sigma) or in fixative without Phalloidin, in which case the egg chambers were stained with 12.5 μg/ml of Phalloidin overnight after four washes in PBS with 0.01% Tween-20. The egg chambers were then washed four times in PBS, 0.01% Tween-20, before mounting in VectaShield mounting media (Vector Laboratories Ltd). As the staining was unstable, confocal images were collected within 24 hr of staining. To compare F-actin levels in different genotypes, we fixed and stained stage 9 oocytes in parallel and imaged the Phalloidin staining of the F-actin 10 μm below the cortex with identical settings. The mean pixel intensity of the images was obtained using Metamorph.

### Drug Treatments

Flies were starved for 2 hr and then allowed to feed on yeast containing 200 μg/ml colcemid (Sigma) for 20 hr or 250 μg/ml latrunculin A (Sigma) for 8 hr before dissection.

## Supplemental Data

Document S1. Four Figures, Five Tables, and Two Movies

Movie S1. A Stage 9 Wild-Type Egg Chamber Expressing Tau-GFP Imaged Once Every 30 Seconds for a Duration of 900 Seconds Green, microtubules; red, yolk autofluorescence (cytoplasm). Posterior is to the right.

Movie S2. Stage 9 *spireRP/Df(2L)Exel6046* Egg Chambers Expressing Tau-GFP Imaged Once Every 30 Seconds for a Duration of 900 Seconds The cytoplasmic flows in the oocyte wash the microtubules into oriented arrays near the oocyte cortex. Green, microtubules; red, yolk autofluorescence (cytoplasm). Both egg chambers are oriented with the oocyte posterior to the right.

## References

[bib1] Alberts A.S. (2001). Identification of a carboxyl-terminal diaphanous-related formin homology protein autoregulatory domain. J. Biol. Chem..

[bib2] Benton R., Palacios I.M., St Johnston D. (2002). *Drosophila* 14–3-3/PAR-5 is an essential mediator of PAR-1 function in axis formation. Dev. Cell.

[bib3] Brendza K.M., Rose D.J., Gilbert S.P., Saxton W.M. (1999). Lethal kinesin mutations reveal amino acids important for ATPase activation and structural coupling. J. Biol. Chem..

[bib4] Cha B.J., Koppetsch B.S., Theurkauf W.E. (2001). In vivo analysis of *Drosophila bicoid* mRNA localization reveals a novel microtubule-dependent axis specification pathway. Cell.

[bib5] Clark I., Giniger E., Ruohola-Baker H., Jan L.Y., Jan Y.N. (1994). Transient posterior localization of a kinesin fusion protein reflects anteroposterior polarity of the *Drosophila* oocyte. Curr. Biol..

[bib6] Cooley L., Verheyen E., Ayers K. (1992). *chickadee* encodes a profilin required for intercellular cytoplasm transport during *Drosophila* oogenesis. Cell.

[bib7] Doerflinger H., Benton R., Torres I.L., Zwart M.F., St Johnston D. (2006). *Drosophila* anterior-posterior polarity requires actin-dependent PAR-1 recruitment to the oocyte posterior. Curr. Biol..

[bib8] Emmons S., Phan H., Calley J., Chen W., James B., Manseau L. (1995). *cappuccino*, a *Drosophila* maternal effect gene required for polarity of the egg and embryo, is related to the vertebrate limb deformity locus. Genes Dev..

[bib9] Eng C.H., Huckaba T.M., Gundersen G.G. (2006). The formin mDia regulates GSK3β through novel PKCs to promote microtubule stabilization but not MTOC reorientation in migrating fibroblasts. Mol. Biol. Cell.

[bib10] Evangelista M., Blundell K., Longtine M.S., Chow C.J., Adames N., Pringle J.R., Peter M., Boone C. (1997). Bni1p, a yeast formin linking cdc42p and the actin cytoskeleton during polarized morphogenesis. Science.

[bib11] Evangelista M., Pruyne D., Amberg D.C., Boone C., Bretscher A. (2002). Formins direct Arp2/3-independent actin filament assembly to polarize cell growth in yeast. Nat. Cell Biol..

[bib12] Fernandez-Borja M., Janssen L., Verwoerd D., Hordijk P., Neefjes J. (2005). RhoB regulates endosome transport by promoting actin assembly on endosomal membranes through Dia1. J. Cell Sci..

[bib13] Frydman H.M., Spradling A.C. (2001). The receptor-like tyrosine phosphatase Lar is required for epithelial planar polarity and for axis determination within *Drosophila* ovarian follicles. Development.

[bib14] Gasman S., Kalaidzidis Y., Zerial M. (2003). RhoD regulates endosome dynamics through Diaphanous-related Formin and Src tyrosine kinase. Nat. Cell Biol..

[bib15] Glotzer J.B., Saffrich R., Glotzer M., Ephrussi A. (1997). Cytoplasmic flows localize injected *oskar* RNA in *Drosophila* oocytes. Curr. Biol..

[bib16] Goode B.L., Eck M.J. (2007). Mechanism and function of formins in the control of actin assembly. Annu. Rev. Biochem..

[bib17] Hwang E., Kusch J., Barral Y., Huffaker T.C. (2003). Spindle orientation in *Saccharomyces cerevisiae* depends on the transport of microtubule ends along polarized actin cables. J. Cell Biol..

[bib18] Johnstone O., Lasko P. (2001). Translational regulation and RNA localization in *Drosophila* oocytes and embryos. Annu. Rev. Genet..

[bib19] Kerkhoff E., Simpson J.C., Leberfinger C.B., Otto I.M., Doerks T., Bork P., Rapp U.R., Raabe T., Pepperkok R. (2001). The Spir actin organizers are involved in vesicle transport processes. Curr. Biol..

[bib20] Kovar D.R., Pollard T.D. (2004). Insertional assembly of actin filament barbed ends in association with formins produces piconewton forces. Proc. Natl. Acad. Sci. USA.

[bib21] Magie C.R., Meyer M.R., Gorsuch M.S., Parkhurst S.M. (1999). Mutations in the Rho1 small GTPase disrupt morphogenesis and segmentation during early *Drosophila* development. Development.

[bib22] Manseau L., Calley J., Phan H. (1996). Profilin is required for posterior patterning of the *Drosophila* oocyte. Development.

[bib23] Manseau L.J., Schupbach T. (1989). *cappuccino* and *spire*: two unique maternal-effect loci required for both the anteroposterior and dorsoventral patterns of the *Drosophila* embryo. Genes Dev..

[bib24] Martin S.G., St Johnston D. (2003). A role for *Drosophila* LKB1 in anterior-posterior axis formation and epithelial polarity. Nature.

[bib25] Martin S.G., McDonald W.H., Yates J.R., Chang F. (2005). Tea4p links microtubule plus ends with the formin For3p in the establishment of cell polarity. Dev. Cell.

[bib26] Minin A.A., Kulik A.V., Gyoeva F.K., Li Y., Goshima G., Gelfand V.I. (2006). Regulation of mitochondria distribution by RhoA and formins. J. Cell Sci..

[bib27] Neuman-Silberberg F., Schüpbach T. (1993). The *Drosophila* dorsoventral patterning gene *gurken* produces a dorsally localized RNA and encodes a TGFα-like protein. Cell.

[bib28] Otomo T., Otomo C., Tomchick D.R., Machius M., Rosen M.K. (2005). Structural basis of Rho GTPase-mediated activation of the formin mDia1. Mol. Cell.

[bib29] Otto I.M., Raabe T., Rennefahrt U.E., Bork P., Rapp U.R., Kerkhoff E. (2000). The p150-Spir protein provides a link between c-Jun N-terminal kinase function and actin reorganization. Curr. Biol..

[bib30] Palacios I.M., St Johnston D. (2002). Kinesin light chain-independent function of the Kinesin heavy chain in cytoplasmic streaming and posterior localisation in the *Drosophila* oocyte. Development.

[bib31] Palazzo A.F., Cook T.A., Alberts A.S., Gundersen G.G. (2001). mDia mediates Rho-regulated formation and orientation of stable microtubules. Nat. Cell Biol..

[bib32] Presgraves D.C. (2003). A fine-scale genetic analysis of hybrid incompatibilities in *Drosophila*. Genetics.

[bib33] Pruyne D., Evangelista M., Yang C., Bi E., Zigmond S., Bretscher A., Boone C. (2002). Role of formins in actin assembly: nucleation and barbed-end association. Science.

[bib34] Quinlan M.E., Heuser J.E., Kerkhoff E., Mullins R.D. (2005). *Drosophila* Spire is an actin nucleation factor. Nature.

[bib35] Rosales-Nieves A.E., Johndrow J.E., Keller L.C., Magie C.R., Pinto-Santini D.M., Parkhurst S.M. (2006). Coordination of microtubule and microfilament dynamics by *Drosophila* Rho1, Spire and Cappuccino. Nat. Cell Biol..

[bib36] Sagot I., Rodal A.A., Moseley J., Goode B.L., Pellman D. (2002). An actin nucleation mechanism mediated by Bni1 and profilin. Nat. Cell Biol..

[bib37] Serbus L.R., Cha B.J., Theurkauf W.E., Saxton W.M. (2005). Dynein and the actin cytoskeleton control kinesin-driven cytoplasmic streaming in Drosophila oocytes. Development.

[bib38] Shulman J.M., Benton R., St Johnston D. (2000). The *Drosophila* homolog of *C. elegans* PAR-1 organizes the oocyte cytoskeleton and directs *oskar* mRNA localization to the posterior pole. Cell.

[bib39] St Johnston D. (2005). Moving messages: the intracellular localization of mRNAs. Nat. Rev. Mol. Cell Biol..

[bib40] Theurkauf W.E. (1994). Premature microtubule-dependent cytoplasmic streaming in *cappuccino* and *spire* mutant oocytes. Science.

[bib41] Theurkauf W.E., Smiley S., Wong M.L., Alberts B.M. (1992). Reorganization of the cytoskeleton during *Drosophila* oogenesis: implications for axis specification and intercellular transport. Development.

[bib42] Van Doren M., Williamson A.L., Lehmann R. (1998). Regulation of zygotic gene expression in *Drosophila* primordial germ cells. Curr. Biol..

[bib43] van Eeden F., St Johnston D. (1999). The polarisation of the anterior-posterior and dorsal-ventral axes during *Drosophila* oogenesis. Curr. Opin. Genet. Dev..

[bib44] Verheyen E.M., Cooley L. (1994). Profilin mutations disrupt multiple actin-dependent processes during *Drosophila* development. Development.

[bib45] Wellington A., Emmons S., James B., Calley J., Grover M., Tolias P., Manseau L. (1999). Spire contains actin binding domains and is related to ascidian posterior end mark-5. Development.

[bib46] Yarmola E.G., Bubb M.R. (2006). Profilin: emerging concepts and lingering misconceptions. Trends Biochem. Sci..

