# Supplementary material for: Capu and Spire Assemble a Cytoplasmic Actin Mesh that Maintains Microtubule Organization in the Drosophila Oocyte
Source: Dev Cell. 2007 Oct 9;13(4):539–53. doi: 10.1016/j.devcel.2007.09.003 (PMC2034408; doi:10.1016/j.devcel.2007.09.003)
Supplement: Document S1. Four Figures, Five Tables, and Two Movies [file mmc1.pdf]

Developmental Cell *13*

Supplemental Data

Capu and Spire Assemble a Cytoplasmic Actin Mesh that Maintains Microtubule Organization in the *Drosophila* Oocyte

Katja Dahlgaard, Alexandre A.S.F. Raposo, Teresa Niccoli, and Daniel St Johnston

Table S1. Frequency of Cytoplasmic Seething and Streaming at Stage 9

Genotype	No. oocytes with cytoplasmic seething	No. oocytes with cytoplasmic streaming
wt	3	0
capu^{G7}*/Df(2l)ed*^{SZ1}	0	15
capu^{G7}*/Df(2l)ed*^{SZ1}*; GFP-Capu*	13	0
capu^{G7}*/Df(2l)ed*^{SZ1}*; GFP-CapuΔN*	10	0
capu^{G7}*/Df(2l)ed*^{SZ1}*; GFP-SpireD*	0	5
spire^{RP}	0	10
spire^{RP}*; GFP-Capu*	5	0
spire^{RP}*; GFP-CapuΔN*	7	0
spire^{RP}*/spire*^{2F}*; GFP-CapuΔN*	8	1
spire^{RP}*/Df(2L)Exel*^{6046}	0	12
spire^{RP}*/Df(2L)Exel*^{6046}*; GFP-SpireD*	5	0
spire^{RP}*/Df(2L)Exel*^{6046}*; GFP-SpireC*	0	3
chic^{1320}	9	16
chic^{1320}*/chic*^{221}	0	9
chic^{1320}*/chic*^{221}*; GFP-Capu*	0	10
chic^{1320}*/chic*^{221}*; GFP-CapuΔN*	0	12

Table showing the number of egg chambers of various genotypes that show either the slow random movements in the oocyte cytoplasm called seething (See Figure 1I), or unidirectional fast movements of premature streaming (See Figure 1J). UASp Capu and Spire constructs were expressed under the control of noa-Gal4-VP16.

Table S2. Microtubule Appearance in Stage 9 and 10A Oocytes

Genotype	Stage9		Stage 10A	
	**Cortical arrays**	**N**	**Cortical arrays**	**N**
w	0%	60	0%	17
capu^{G7}*/Df(2l)ed*^{SZ1}	100%	76	100%	40
capu^{G7}*/Df(2l)ed*^{SZ1}; *GFP-Capu*	0%	58	0%	13
capu^{G7}*/Df(2l)ed*^{SZ1}; *GFP-CapuΔN*	0%	65	0%	24
capu^{G7}*/Df(2l)ed*^{SZ1}; *GFP-Spir-D*	100%	27	100%	12
spire^{RP}	99%	95	100%	15
spire^{RP}; *Capu*	48%	83	100%	17
spire^{RP}; *GFP-Capu*	12%	200	56%	32
spire^{RP}; *GFP-CapuΔN*	1%	69	40%	15
spire^{RP}*/spire*^{2F}; *GFP-Spir-D*	0%	32	0%	10
capu^{RK} *FRT nlsGFP/ capu*^{G7}	95%	72	100%	8
capu^{RK} *khc*^{17}*/ capu*^{G7} *khc*^{17}	0%	8	0%	2

Table showing the percentage of egg chambers with dense arrays of microtubules along the oocyte cortex (See Figure 1O) instead of the normal anterior to posterior gradient of microtubules (See Figure 1M). N indicates the number of egg chambers analysed for each genotype. UASp Capu and Spire constructs were expressed under the control of noa-Gal4-VP16.

Table S3. *oskar* mRNA Localisation in Stage 9-10B Egg Chambers

Genotype	Posterior	Posterior & Diffuse	Diffuse	N
W	100%	0%	0%	71
capu^{G7}*/Df(2l)ed*^{SZ1}	0%	0%	100%	56
capu^{G7}*/Df(2l)ed*^{SZ1}; *GFP-CapuΔN*	100%	0%	0%	106
spire^{RP}	0%	2%	98%	101
spire^{RP}; *GFP-Capu*	21%	70%	9%	133
spire^{RP}; *GFP-CapuΔN*	53%	21%	25%	255
spire^{2F}*/spire*^{RP}; *GFP-Spir-D*	100%	0%	0%	26

Table showing the distribution of *oskar* mRNA localisation phenotypes, as visualised by *oskar in situ* hybridisation, in stage 9-10B egg chambers of various genotypes. The localisation patterns were classified as: Diffuse localisation (See Figure 1N); Posterior and diffuse (See Figure S2B) and Posterior localisation (See Figure 1P). N indicates the number of egg chambers analysed for each genotype. UASp Capu and Spire constructs were expressed under the control of noa-Gal4-VP16.

Table S4. The Effect of Latrunculin A on Ooplasmic Movement at Stage 9

Genotype	Without Latrunculin A		With Latrunculin A	
	Streaming	Seething	Streaming	Seething
w	0	3	12	0
capu^{G7}*/Df(2l)ed*^{SZ1}*;GFP-Capu*	0	13	14	1
capu^{G7}*/Df(2l)ed*^{SZ1}*;GFP-CapuΔN*	0	10	0	6
spire^{RP}*/spire*^{2F}*;GFP-Spir-D*			7	0
spire^{RP}*/spire*^{2F}*;GFP-CapuΔN*	1	8	7	0

Table showing the number of control egg chambers or egg chambers treated with Latrunculin A of various genotypes that show either slow random movements of the oocyte cytoplasm (seething (see Figure 1I)), or the fast unidirectional movements of premature streaming (See Figure 1J). UASp Capu and Spire constructs were expressed under the control of noa-Gal4-VP16.

Table S5. The Effect of Latrunculin A on Microtubule Appearance at Stage 9

Genotype	Without Latrunculin A		With Latrunculin A	
	Cortical arrays	N	Cortical arrays	N
w	0%	13	100%	55
capu^{G7}*/Df(2l)ed*^{SZ1}*;GFP-Capu*	0%	54	97%	65
capu^{G7}*/Df(2l)ed*^{SZ1}*;GFP-CapuΔN*	0%	53	0%	54
spire^{RP}*/spire*^{2F}*;GFP-Spir-D*	0%	33	100%	63
spire^{RP}*/spire*^{2F}*;GFP-CapuΔN*	52%	67	96%	56

Table giving the percentage of control stage 9 egg chambers or stage 9 egg chambers treated with Latrunculin A that contain dense arrays of microtubules along the oocyte cortex (See Figure 1O) instead of the normal anterior to posterior gradient of microtubules (See Figure 1M). N indicates the number of egg chambers analysed for each genotype. UASp Capu and Spire constructs were expressed under the control of noa-Gal4-VP16.

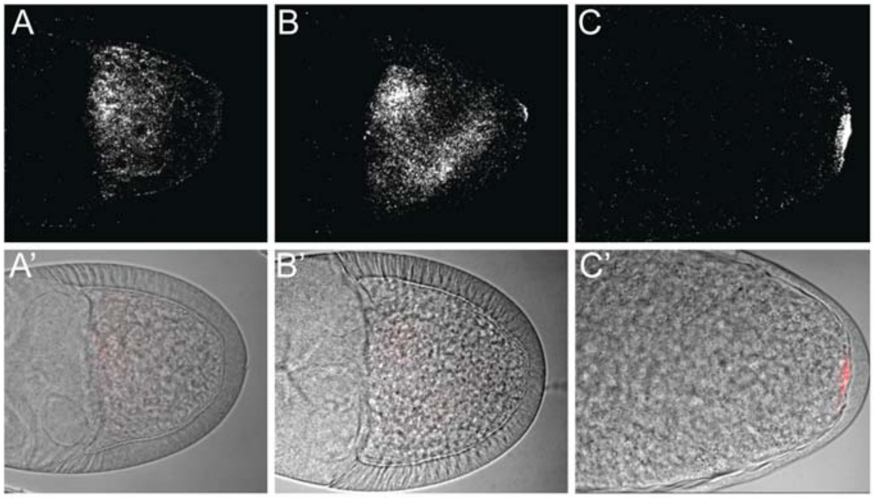

S1 D. *oskar* mRNA localisation in *spire*^{RP}/*Df(2L)Exel*^{6046} egg chambers				
**Stage**	**Diffuse**	**Posterior & Diffuse**	**Posterior**	**N**
9	97%	3%	0%	117
10A	86%	14%	0%	71
10B	68%	32%	0%	66
11-13	46%	50%	4%	54

Figure S1. *oskar* mRNA Localises to the Posterior of Late Stage *spire* Mutant Oocytes

(A-C') Fluorescent in situ hybridisations to *oskar* mRNA in *spire*^{RP}/*Df(2L)Exel*^{6046} egg chambers. (A-C) Shows the fluorescent signal from an in situ hybridization to *oskar* mRNA, while (A'-C') show DIC images of the oocytes. (A and A') Stage 9; (B and B') Stage 10A; (C and C') Stage 13. As oogenesis progresses, more *oskar* mRNA accumulates at the posterior of the oocyte.

(D) Table showing the distribution of *oskar* mRNA at different stages of oogenesis in *spire*^{RP}/*Df(2L)Exel*^{6046} oocytes. The localisation patterns are classified as diffuse localisation as seen in (A); posterior and diffuse as in (B) and posterior as in (C).N indicates the number of egg chambers analysed at each stage.

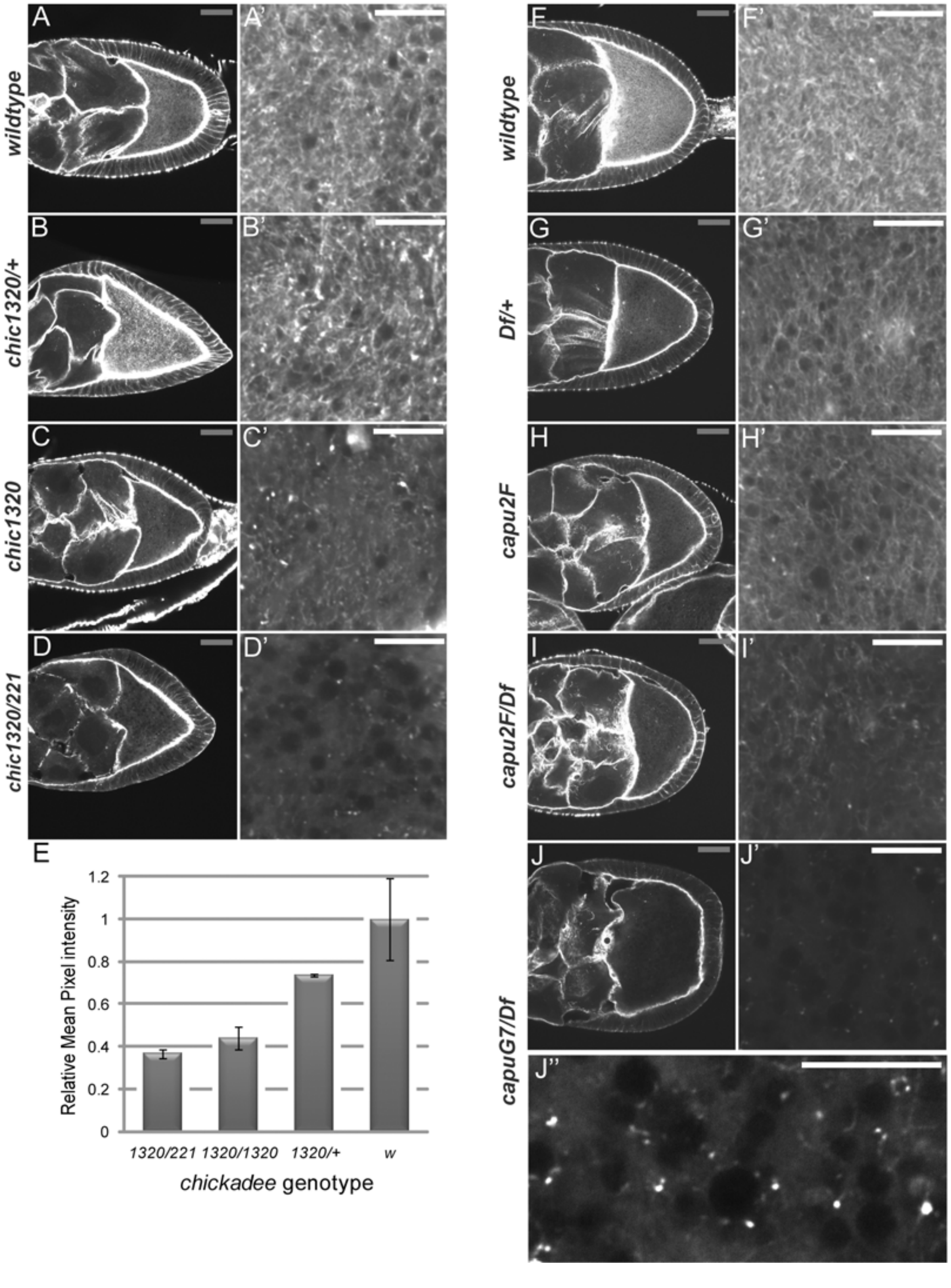

Figure S2. Comparisons of the Actin Mesh in a Range of *chic* and *capu* Alleles

(A-D', F-J') Stage 9 egg chambers stained with TRITC-Phalloidin. (A'-D') show magnifications of the oocyte cytoplasm imaged 10 µm below the cortex. (A-D') and (F-J') show egg chambers stained, imaged and processed in parallel under identical conditions. The vertical lines show the standard deviations in mean pixel intensities. The F-actin forms a dense actin mesh in wildtype oocytes (A). The actin mesh appears slightly less dense in *chic*^{1320}/+ (B), is almost gone in *chic*^{1320} oocytes, (C), and is completely absent in *chic*^{1320}/*chic*^{221} oocytes (D).

(E) Bar diagram showing the relative average of mean pixel intensity of the F-actin staining in 30 µm by 30 µm regions of 3-5 stage 9 egg chambers of different *chic* genotypes.

(F-J’’) A similar graded effect on the actin mesh is observed in *capu* mutant combinations of increasing strength. (F) Wildtype; (G) *Df(2L)ed*^{SZ1}/+ ; (H) *capu*^{2F}; (I) *capu*^{2F}*/Df(2L)ed*^{SZ1}; (J) *capu*^{G7}*/Df(2L)ed*^{SZ1} . (J’’) shows the bright dots of residual F-actin staining in the oocyte cytoplasm in the absence of Capu.

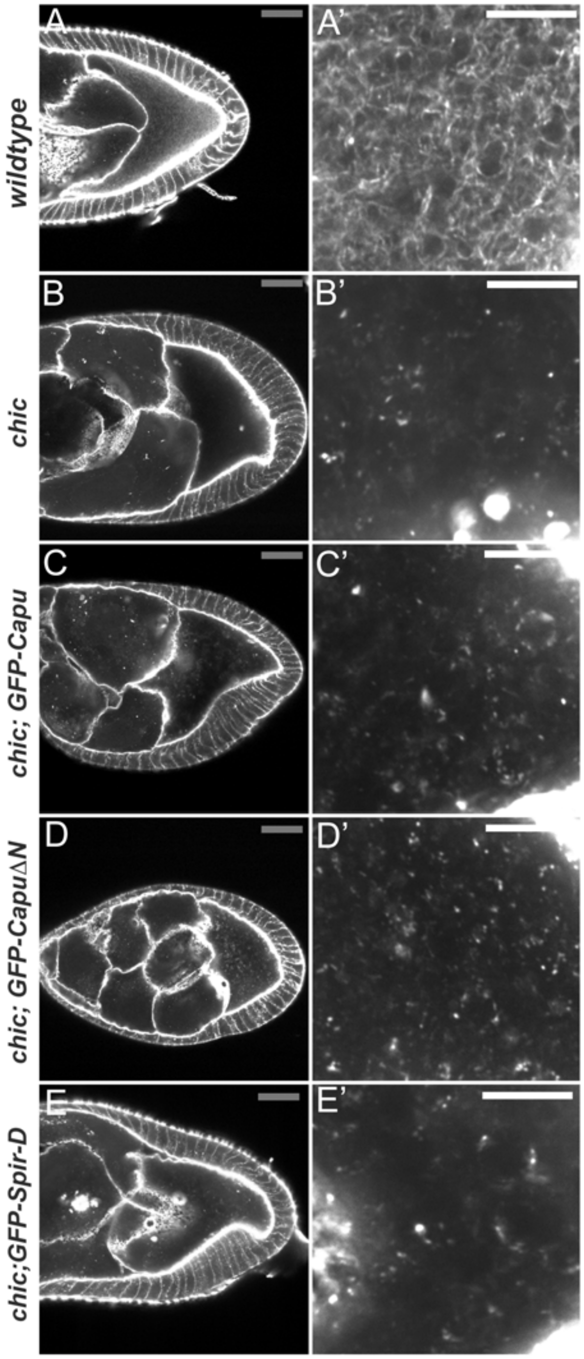

Figure S3. The *chic* Mutant Phenotype Is Not Rescued by GFP-Capu, GFP-CapuΔN, or GFP-SpireD

(A-E) Confocal images of s7age 8-9 egg chambers stained with TRITC-Phalloidin to label F-actin. (A’- E’) show a magnification of the oocyte cytoplasm imaged 10 µm from the cortex.

(A) wildtype egg chamber, (B) *chic*^{1320}/*chic*^{221}, (C) *chic*^{1320}/*chic*^{221}; *GFP-Capu*, (D) *chic*^{1320}/*chic*^{221}; *GFP-CapuΔN*, (E) *chic*^{1320}/*chic*^{221}; *GFP-Spir-D*.

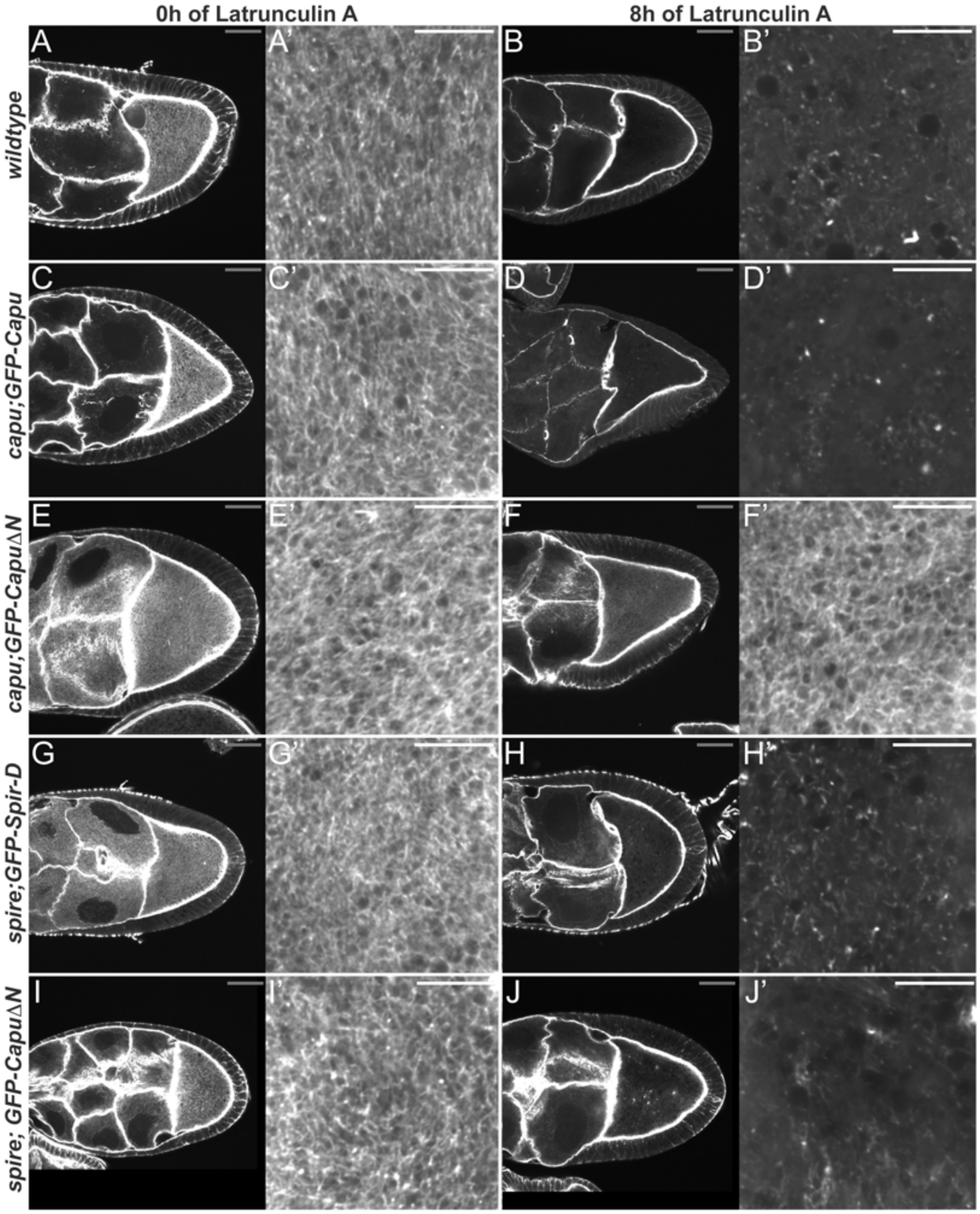

Figure S4. The Actin Mesh in Untreated and Latrunculin A Treated Egg Chambers

(A-J) Confocal images of stage 9 egg chambers stained with TRITC-Phalloidin to label F-actin, (Grey scale bar, 30 µm). (A’-J’) show a magnification of the F-actin stained oocyte cytoplasm imaged 10 µm from the cortex (White scale bar, 10µm). (A,C,E,G, I) are images of untreated egg chambers, while (B,D,F,H) are images of egg chambers after 8 hours of treatment. (A-D) were processed in parallel as were (E) and (F), (G) and (H), and (I) and (J).

(A-B’) wildtype; (C-D’) *capu*^{G7}/*Df(2L)ed*^{SZ1} ; *GFP-Capu*; (E-F’) *capu*^{G7}/*Df(2L)ed*^{SZ1} ; *GFP-CapuΔN*; (G-H’) *spire*^{2F}/*spire*^{RP}; *GFP-Spir-D*; (I-J’) *spire*^{2F}/*spire*^{RP}; *GFP-CapuΔN*.
